# Placental thrombomodulin expression in recurrent miscarriage

**DOI:** 10.1186/1477-7827-8-1

**Published:** 2010-01-05

**Authors:** Piergiorgio Stortoni, Monia Cecati, Stefano R Giannubilo, Davide Sartini, Angelo Turi, Monica Emanuelli, Andrea L Tranquilli

**Affiliations:** 1 Department of Clinical Sciences, Polytechnic University of Marche, Ancona, Italy; 2 Department of Biochemistry Biology and Genetics, Polytechnic University of Marche, Ancona, Italy

## Abstract

**Background:**

Early pregnancy loss can be associated with trophoblast insufficiency and coagulation defects. Thrombomodulin is an endothelial-associated anticoagulant protein involved in the control of hemostasis and inflammation at the vascular beds and it's also a cofactor of the protein C anticoagulant pathway.

**Discussion:**

We evaluate the Thrombomodulin expression in placental tissue from spontaneous recurrent miscarriage and voluntary abortion as controls. Thrombomodulin mRNA was determined using real-time quantitative polymerase chain reaction. Reduced expression levels of thrombomodulin were found in recurrent miscarriage group compared to controls (1.82-fold of reduction), that corresponds to a reduction of 45% (from control group Delta CT) of thrombomodulin expression in spontaneous miscarriage group respect the control groups.

**Summary:**

We cannot state at present the exact meaning of a reduced expression of Thrombomodulin in placental tissue. Further studies are needed to elucidate the biological pathway of this important factor in the physiopathology of the trophoblast and in reproductive biology.

## Background

Thrombomodulin (TM) is a glycoprotein receptor composed of 559 amino acid residues and expressed mainly on the endothelial surface of blood vessels and in the placental syncytiotrophoblast [1]. It is composed of different structural domains: an N-terminal lectin-like module, a hydrophobic region, six EGF-like repeats, a Ser/Thr-rich region, a trans-membrane region and a short cytoplasmic tail [2]. This glycosylated transmembrane receptor for activated coagulation Factor IIa [3] in the intact endothelium thrombomodulin forms a complex with thrombin which is responsible of converting protein C to activated protein C [1,4,5]. The Thrombomodulin-thrombin complex balances the substrate specificity of thrombin, erasing its procoagulant properties, and improving the conversion of protein C into its activated form [6]. Protein S and activated protein C cleave in a proteolytic way the activated coagulation factors Va and VIIIa: all this complex process inactivates the enzymatic complexes that make coagulation factors Xa and thrombin active. If the blood coagulation inhibitor Thrombomodulin lacks from trophoblast cells of the mouse placenta, a fatal arrest of placental morphogenesis occurs [7], leading to embryo failure probably due to tissue factor-initiated activation of the blood coagulation cascade at the fetus-maternal interface [8]. In addition, TM integrates fibrinolytic and anti-inflammatory responses also independently by protein C and thrombin. The analysis of different mouse models has revealed novel and in part organ-specific functions of TM, most notably in the vascular bed of the placenta. In these mouse models, the severity and phenotypic expression of thrombosis is highly variable and is dependent on the interaction with secondary genetic or environmental modifiers. The mutant mouse strains replicate important aspects of thrombophilia and thrombosis in humans [9].

The functions of TM are exerted in two distinct tissues: in non-endothelial extra-embryonic tissues, required for proper function of the early placenta, in embryonic blood vessel endothelium whose absence causes lethal consumptive coagulopathy [10].

## Discussion

With this background, we performed a prospective case-control study measuring the gene expression of thrombomodulin in placental tissues from spontaneous recurrent miscarriage and voluntary abortion by Real-Time quantitative PCR to elucidate the role of this factor on human miscarriage.

Trophoblastic tissue was obtained during surgical uterine evacuation in 11 women with recurrent miscarriage and 20 healthy primiparous pregnant women undergoing elective termination of pregnancy matched for gestational age. Recurrent miscarriage was defined as occurrence of three consecutive spontaneous miscarriages before 20 weeks' gestation [11]. In the group with recurrent miscarriage were recruited only the patients with incoming miscarriage (< 12 hours) who were admitted in hospital for threatened abortion.

All women enrolled were regularly menstruating, with a cycle length of < 35 days. Clinical details were recorded for each woman, and only patients who were certain of their menstrual dates entered the study group. Specific exclusion criteria for all women enrolled included conception by assisted reproduction, gestational diabetes, diabetes mellitus, a history of smoking and hypertension, proteinuria, renal, cardiovascular hepatic, and endocrine disease, metabolic disorders, and current infection or history of all types of infection. Moreover, according to our internal protocol, enrolled subjects did not present with thyroid disease, congenital or acquired thrombophilic disorders, or chromosomal or other fetal and uterine anomalies.

None of these women had non-obstetric clinical features of antiphospholipid syndrome (i.e., history of thrombosis, autoimmune thrombocytopenia, systemic lupus erythematosus, or other autoimmune conditions). All had negative test results for lupus anticoagulant and fewer than 20 IgG phospholipid-binding units of anticardiolipin antibodies. Furthermore, none of the recruited women took any regular medication, which included antihypertensive drugs or MgSO4, before and during the experiment, and none of the control subjects were on oral contraception; only folic acid (400 μg/day) was taken during pregnancy and diets were comparable for all groups.

Since in first trimester placenta a number of other cell types present within the villous stroma such as fibroblasts, myofibroblasts, smooth muscle cells, pericytes and endothelial cells as well as maternal and fetal blood cells are potential contaminants, the tissues derived by curettage were characterized using antibodies to cytokeratin antibodies specific for the isoform 7 to obtain an high grade of trophoblast purification [12].

**Figure 1:**
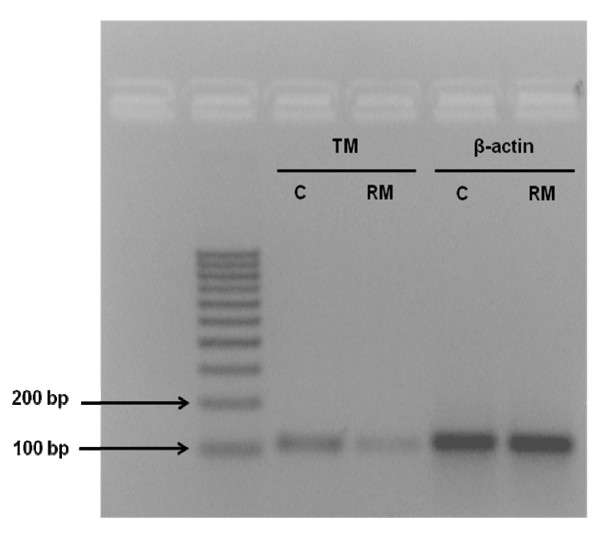
**Real-time PCR analysis**. TM and β-actin amplicons from RM (recurrent miscarriage) and C (controls) were analyzed by electrophoresis on 2% agarose gel.

**Figure 2:**
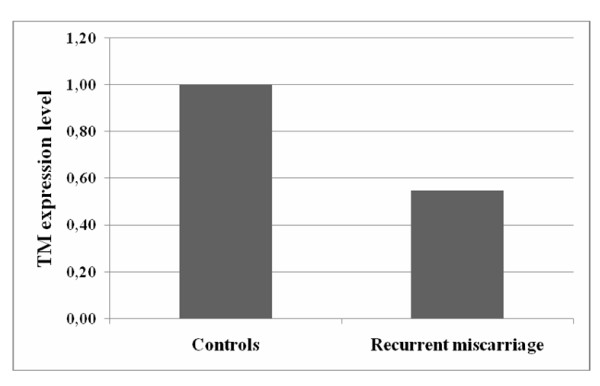
**Thrombomodulin expression is significantly reduced in spontaneous recurrent miscarriage versus control group (p < 0.05)**.

All specimens were snap-frozen in liquid nitrogen and stored at -80°C until use. The total length of processing time was less than 10 minutes. The study was performed in accordance with the principles of the Declaration of Helsinki as revised in 2001. The study was approved by the local ethics committee, and informed consent was given by all women enrolled in the study. A piece of the frozen tissue (20-40 mg) was homogenized in lysis buffer, and the total RNA was extracted with an RNA isolation kit (Promega, Madison, WI). RNA samples were tested by ultraviolet absorption at 260 nm in order to determine RNA concentration. The quality and concentration of the RNA samples were further confirmed by electrophoresis on denaturated 1% agarose gels. Two micrograms of RNA were reverse transcribed in a total volume of 25 μl for 60 min at 37°C with M-MLV Reverse Transcriptase (Promega, Madison, WI) using random nonamers in order to obtain complementary DNA (cDNA). cDNA was used for real-time quantitative PCR. To avoid false-positive results attributable to the amplification of contaminating genomic DNA in the cDNA preparation, the primers were selected to flank an intron and PCR efficiencies were tested and found to be close to 1. The following primers were used: 5'CTCTTCCAGCCTTCCTTCCT-3' (forward), 5'-AGCACTGTGTTGGCGTACAG-3' (reverse) for β-actin, and 5'-TAACGAAGACACAGACTGCGATT-3' (forward) and 5'-ACCTTGACCTCGTGGGCTAG 3' (reverse) for thrombomodulin. The genes were run in duplicate using SYBR Green chemistry. All samples, taken from each pregnancy, were tested in triplicate using as reference gene β-actin for data normalization to correct for variations in RNA quality and quantity. To increase accuracy of normalization, a second housekeeping gene (GAPDH) was used. No significant differences were observed in thrombomodulin expression levels with the use of the additional endogenous control. Direct detection of the PCR products was monitored by measuring the fluorescence produced by SYBR Green I dye binding to the double-strand DNA after every cycle. These measurements were then plotted against cycle numbers. The parameter *Ct *(threshold cycle) was defined as the cycle number at which the first detectable fluorescence signal above the threshold was observed. The real-time PCR products were also electrophoresed on 2% agarose gel, and the intensity of amplicons of expected size was evaluated (Figure 1). Fold changes in relative gene expression were calculated by 2^-Δ(Δ*Ct*) ^where Δ*Ct *= *Ct *(thrombomodulin) - *Ct *(β-actin) and Δ(Δ*Ct*) = mean ΔCt (pathological placentas) - mean Δ*Ct *(normal placentas). The Mann-Whitney U test was performed to compare data from control and pathological placentas. Differences were considered significant at p < 0.05. The results were obtained as "fold changes" in relative gene expression of study group respect to controls. All values were expressed as Mean ΔCt ± Standard Deviation, Median ΔCt and first and third Quartile. The two groups were comparable for maternal age and gestational age, the characteristics of the patients recruited are reported in the Table 1. Reduced expression levels of thrombomodulin were found in recurrent miscarriage group compared to controls (1,82-fold reduction), that corresponds to a reduction of 45% (from control group ΔCt) of thrombomodulin expression in spontaneous miscarriage group respect the control group (Figure 2).

**Table 1:** Gestational age and Thrombomodulin (TM) expression level in the two groups

	Controls (N = 20)	Recurrent Miscarriage (N = 11)	p <
**Age (yrs)**	32.6 ± 2.1	33.2 ± 2.9	N.S.
**Gestational Age (weeks)**	10.1 ± 1.91	10.9 ± 1.56	N.S.
**TM ΔCt (Median)**	6.410	7.133	< 0.05
**TM ΔCt (Mean ± SD)**	6.377 ± 0.213	7.230 ± 0.870	< 0.05
**First quartile**	6,14	6,77	< 0.05
**Third quartile**	6.59	7.95	< 0.05

Thrombomodulin is considered a protein involved in coagulation, cancer and embryogenesis. The altered coagulation process leads to a precocious placental failure, but some studies underline that Thrombomodulin has an anti-inflammatory ability through both protein C dependent and independent pathways. The activation of the coagulation cascade may result in a positive-feedback loop consisting of thrombin-mediated repression of Thrombomodulin-dependent protein C activation [13]: this aspect shows the strict relation between coagulation and inflammation, two processes that enhance one to each other leading to the early placental vascular damage. In pregnancy, fibrinolysis is controlled by the maternal endothelium and placenta, both of which are central to the pathogenesis of some obstetric pathologies [14]. Some works have discussed that plasma Thrombomodulin levels might point out placental vascular endothelial damage reflecting on birthweight [15]. On the contrary, other studies have focused the attention on the role of the Thrombomodulin in cell invasivity and capability to perform intravasation and extravasation, during local invasion.

In this work we found a reduced expression of Thrombomodulin in placental tissue of women who experienced spontaneous recurrent abortion by Real-Time quantitative PCR.

This result confirms the observations that loss of function of Thrombomodulin causes early post-implantation embryonic lethality [16], underlying that Thrombomodulin expression in non-endothelial placental cells is required for a normal function of the early placenta. By this hypothesis the absence of Thrombomodulin from blood vessel endothelium may cause excessive activation of the embryonic blood coagulation system [17]. We cannot state at present the exact meaning of a reduced expression of Thrombomodulin in placental tissue. Further studies are needed to elucidate the biological pathway of this important factor in the physiopathology of the trophoblast and in reproductive biology.

## Competing interests

The authors declare that they have no competing interests.

## Authors' contributions

PS, SRG, ME, AT and ALT conceived of the study, and participated in its design and coordination and helped to draft the manuscript, MC carried out the molecular genetic studies, participated in the sequence alignment and drafted the manuscript. All authors read and approved the final manuscript.
